# The frequency of assessment of progression in randomized oncology clinical trials

**DOI:** 10.1002/cnr2.1527

**Published:** 2021-11-24

**Authors:** Alyson Haslam, Jennifer Gill, Vinay Prasad

**Affiliations:** ^1^ Department of Epidemiology and Biostatistics University of California San Francisco California USA; ^2^ Providence Health & Services Portland Oregon USA

**Keywords:** clinical trial, progression, tumor assessment

## Abstract

**Background:**

Progression in tumor assessments is often detected at a follow‐up appointment rather than when actual change in progression has occurred, which can bias PFS outcomes.

**Aim:**

We sought to evaluate the frequency of tumor assessment scans in clinical trials of anti‐cancer interventions and to compare this to recommended (National Comprehensive Cancer Network) and real‐world frequencies of tumor assessments.

**Methods:**

In a cross‐sectional analysis, we searched for articles published in the three top oncology journals between July 2017 and June 2020. We included articles that were RCTs of patients that had unresectable or metastatic solid tumors and used an intervention that was designed to be anti‐tumor. We abstracted median PFS survival for each group, the PFS hazard ratio, frequency of tumor assessment scans, tumor type, intervention type, and information regarding the study.

**Results:**

We found that, in the 182 comparisons (163 articles), less frequent tumor assessment (occurring more than 9 weeks between assessments) was associated with higher median PFS values for both the intervention group (*p* < .0001) and the control group (*p* < .0001). PFS hazard ratios for studies scanning for tumors every 10 or more weeks were no different than for studies scanning for tumors more frequently (*p* = .88). Data on the frequency of tumor assessments in the real world is sparse.

**Conclusion:**

We found that less frequent tumor assessment frequency was associated with longer median PFS in both intervention and control groups of clinical oncology trials but was not associated with differences in PFS hazard ratios. Future research is needed to compare real world to trial assessment.

## 1 BACKGROUND

Progression‐free survival (PFS) is one of the most common primary endpoints in oncology clinical trials,^1^ and is a composite of death or tumor growth after treatment. Because the measurement of PFS primarily relies on tumor assessment, it can be biased by variables such as the timing and methodology of tumor assessment. As such, the increasing use of PFS in place of other well‐established outcomes, such as overall survival, in oncology trials should rely on measurements with minimal bias and consistent protocols.

Previous authors have suggested several recommendations for designing and executing clinical trials in reducing bias related to PFS outcomes measurement, including assessment bias (related to the frequency of tumor assessments) and evaluation bias (treatment arms receiving tumor assessments at different frequencies).^2^ The timing of tumor assessments is especially influential on PFS outcomes since progression is often detected at a follow‐up appointment rather than when actual change in progression has occurred,^3^ thus a longer time interval between tumor assessments can overestimate PFS.

An additional consideration is whether the tumor assessment in clinical trials is representative of real‐world practice, or at least practices that are recommended for the real‐world practice. The US National Comprehensive Cancer Network has issued recommended scan frequencies for some cancers, and the frequency of scans varies by cancer type and treatment type, recommending every 2–6 months for women with breast cancer treated with endocrine therapy and every 6–12 weeks for women treated with cytotoxic chemotherapy, and as frequently as every 6–16 weeks for kidney cancer.^4^

It is with this background that we sought to evaluate the frequency of tumor assessment scans in clinical trials of anti‐cancer interventions for solid tumors and to compare this to recommended and real‐world frequencies of tumor assessments. We further sought to assess the association between tumor assessment frequency and PFS measurements.

## 2 METHODS

We sought to characterize the frequency of tumor assessments in the literature and to see if there is an association between tumor assessment frequency and either PFS or overall survival indices in oncology studies.

### 2.1 Article inclusion and data abstraction

Articles published in the three top oncology journals (Lancet Oncology, JAMA Oncology, and Journal of Clinical Oncology) between July 2017 and June 2020 were considered for inclusion. We included articles that were RCTs of patients with cancers that were unresectable or metastatic solid tumors and used an intervention that was designed to be anti‐tumor. Studies that did not report PFS hazard ratio, median PFS, or tumor assessment frequency, were pooled analyses, were dose‐optimization studies (no comparator), or the intervention was to prevent cancer were excluded. For studies that had more than two arms, we analyzed each comparison separately.

We abstracted median PFS survival for each group, the PFS hazard ratio, frequency of tumor assessment scans, tumor type, intervention type, whether the tumor assessments were blinded (double blind or masked tumor assessment vs. open and unmasked tumor assessments), and information regarding the study (e.g., publication date, journal, etc.). Because a number of studies did not report a PFS hazard ratio, we calculated a risk ratio from the reported median PFS control and intervention values for all studies. Both the risk ratio and the PFS hazard ratio were used as separate outcomes. For the three studies where median PFS was not reached, we used the time the study participants were followed in place of the median PFS. Most studies assessed the tumor response at regular intervals, but for those that had varying frequencies, we used the frequency first used lasting 6 months or longer. We then categorized tumor assessment frequency by <8 weeks, 8–9 weeks, and >9 weeks.

We then compared the tumor assessment frequency with guideline recommendations (National Comprehensive Cancer Network) for the frequency of tumor assessments. We also searched on Google Scholar and PubMed for studies that reported on the frequency of tumor assessment in real‐world clinical practice. For search terms, we used the cancer type (for the five most common) and “frequency of tumor assessment real world”. For this search, we did not include articles that reported frequency in clinical trials.

### 2.2 Statistical analysis

We calculated descriptive statistics for included studies. We calculated differences in PFS and overall survival indices by tumor assessment frequency category using analysis of covariance. We ran separate models for PFS hazard ratio, PFS risk ratio, and overall survival hazard ratio. To examine the effects of blinding and tumor type on PFS outcomes, we calculated differences in PFS indices by tumor assessment frequency, stratified by whether the study was blinded or not and by the most common tumor types. We did not include one study in the model analyses because it was found to be an outlier when we checked model residuals. We checked model assumptions by using a QQ plot for normality and the residuals versus fits plot for homogeneity of variance. All data were publicly available and non‐identifiable to patients or study participants, so no institutional review board approval was required. All analyses were done using R statistical software.

## 3 RESULTS

We reviewed 1484 articles. Among excluded articles, 947 were not RCTs; 171 trials did not include patients with unresectable or metastatic cancer; 54 did not include PFS outcomes; 55 used interventions that were not anti‐tumor; 40 involved non‐solid tumors; 26 were subgroup or secondary analyses; 17 were pooled analyses; 6 had interventions designed to prevent cancer (not treat), and one was a dose‐finding study. We further excluded two articles ‐ one article was retracted, and another article reported that the median PFS was not reached in either arm and did not report a hazard ratio, and therefore we had no numbers to use in the analysis. We then excluded two additional studies because they did not report a tumor assessment frequency. The remaining 163 articles were included in the data analysis. Sixteen articles had multiple arms, which resulted in 182 total comparisons.

The most common cancers studied were: non‐small cell lung cancer (*n* = 29 studies; 18%); breast (*n* = 23 studies; 14%); colorectal cancer (*n* = 14 studies; 8%); prostate (*n* = 12 studies; 7%); ovarian (*n* = 12 studies; 7%); melanoma (*n* = 8 studies; 5%); and gastric (*n* = 7; 4%). Most studies were phase 3 (*n* = 90; 54%), followed by phase 2 (*n* = 68; 42%), phase 4 (*n* = 1; 1%) and phase 1 (*n* = 2; 1%), with three studies not indicating the phase (2%). Ninety‐seven studies (60%) did not use a blinded tumor assessment, while 66 studies (40%) did.

The percentage of studies with blinded tumor assessments by tumor assessment category was as follows: 38% of studies had tumor assessments being done every 12 weeks or longer; 36% studies had tumor assessments being done every 8 weeks; and 45% of studies had tumor assessments done less than every 8 weeks (chi‐square = 0.14; *p* = .93).

**FIGURE 1:**
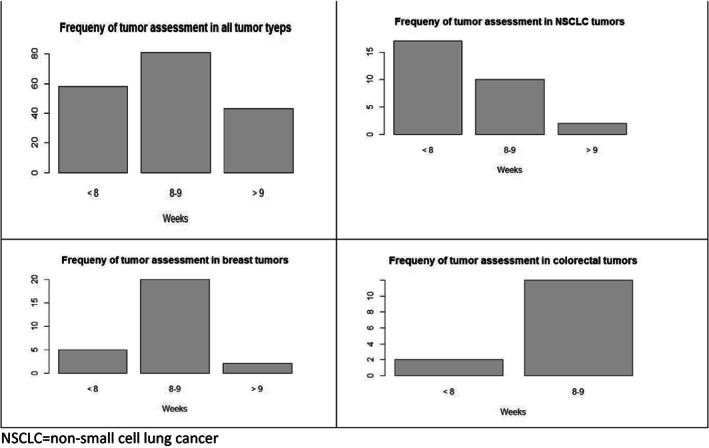
Frequency of tumor scans in oncology studies assessing progression free survival in all tumor types combined (overall) and the 3 most common cancer types encountered in studies published in the top 3 oncology journals July 2017 through June 2020. NSCLC, non‐small cell lung cancer

The median scan frequency for the studies was every 8 weeks (range 4–24 weeks; 41 studies with tumor assessments being done every 12 weeks or longer; 64 studies with tumor assessments being done every 8 weeks and 58 studies conducting tumor assessments less than every 8 weeks). Figure 1 shows the frequency of tumor assessments for all tumor types combined and the three most common cancers reported on in our analysis. The median PFS for the 182 intervention and control groups were 7 and 5.4 months, respectively. The median hazard ratio was 0.74.

**FIGURE 2:**
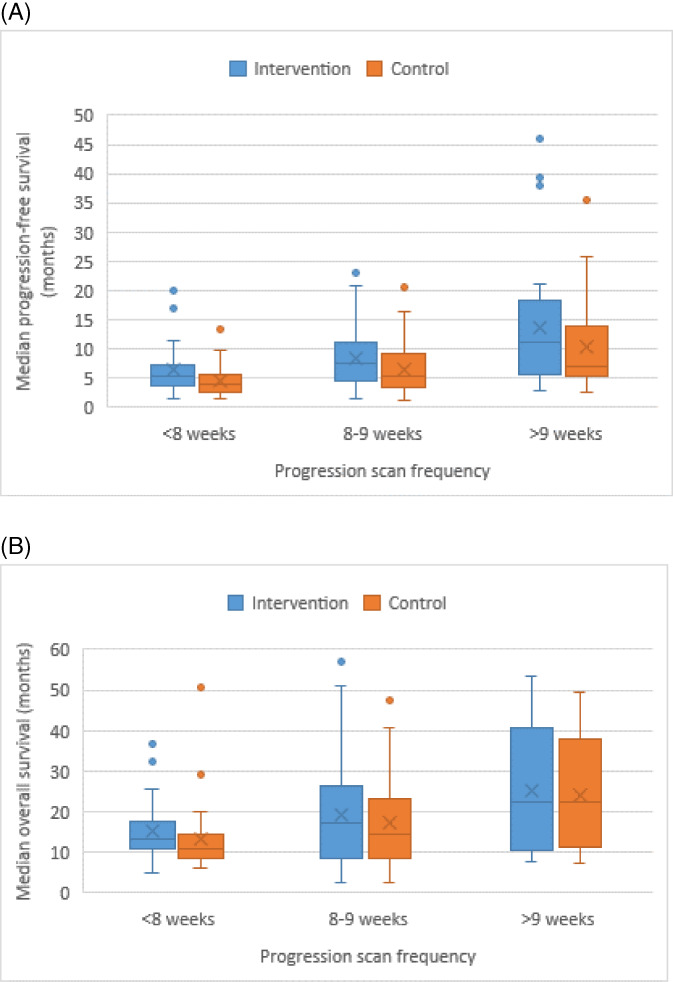
Box and whisker plot of progression‐free survival (A) or overall survival (B) and progression scan frequency in randomized studies of solid‐tumor anti‐cancer therapies

We found that less frequent tumor assessment (occurring more than 9 weeks between assessments) was associated with higher median PFS values for both the intervention group (F = 21.76; *p* < .0001; Table 1 and Figure 2) and the control group (F = 25.24; *p* < .0001). Median overall survival times were higher for studies that had tumor assessment every 10 or more weeks for both the intervention group (F = 7.91; *p* < .0001; Table 1 and Figure 2) and the control group (F = 9.00; *p* = .0002). Both PFS (F = 0.13; *p* = .88) and overall survival (F = 1.54; *p* = .22) hazard ratios for studies scanning for tumors every 10 or more weeks were numerically higher than for studies scanning for tumors more frequently, but the differences were not significant (Table 1). Model assumptions for normality and homogeneity of variance were met in these models.

**TABLE 1:** Mean values (and 95% confidence intervals) for progression‐free hazard ratios, median progression‐free survival for the intervention group, median progression‐free survival for the control group, and overall survival hazard ratio, by progression scan frequency for randomized, metastatic oncology studies

	Tumor scan frequency			*p*‐value*
	<8 weeks	8–9 weeks	>9 weeks	
Progression‐free survival risk ratio (*N* = 157)^a^	0.80 (0.71 to 0.88)	0.80 (0.72 to 0.87)	0.84 (0.74 to 0.95)	.73
Progression‐free survival hazard ratio (*N* = 176)^b^	0.73 (0.66 to 0.80)	0.74 (0.69 to 0.80)	0.76 (0.67 to 0.85)	.88
Median progression‐free survival for the intervention group (*N* = 176)	6.12 (4.39 to 7.85)	8.90 (7.45 to 10.34)	14.95 (12.92 to 16.99)	<.0001
Median progression‐free survival for the control group (*N* = 176)	4.47 (3.17 to 5.76)	6.48 (5.39 to 7.56)	11.55 (10.02 to 13.07)	<.0001
Overall survival hazard ratio (*N* = 124)	0.89 (0.82 to 0.96)	0.87 (0.81 to 0.93)	0.97 (0.88 to 1.06)	0.22
Median overall survival for the intervention group (*N* = 124)	14.57 (11.26 to 17.87)	19.82 (16.76 to 22.89)	26.2 (21.31 to 31.09)	<.0001
Median overall survival for the control group (*N* = 124)	13.99 (10.98 to 17.00)	17.22 (14.31 to 20.15)	25.29 (20.95 to 29.62)	.0002

^a^ Simple ratio of median progression‐free survival (PFS) for control group over the median PFS for intervention group, without accounting for censoring and timing of progression.

^b^ Sub analysis that included only studies that reported a PFS hazard ratio.

* Analysis of variance *p*‐value for global differences between groups.

We found that PFS hazard ratios were higher in studies with unblinded assessments than studies that had blinded assessments (HR = 0.79; 95% CI = 0.73 to 0.84 for unblinded vs. HR = 0.68; 95% CI = 0.62 to 0.73 for blinded; *p* = .006; data not shown), but there was no indication of interaction between scan frequency and blinded status of the study (*p* = .16). We did not find any differences in PFS hazard ratio tumor between categories of tumor assessment frequency in either blinded studies or (*p* = .62) or unblinded studies (*p* = .50). When looking at the association between tumor scan frequency and PFS hazard ratio in each of the three most common cancer types, there was no association (NSCLC *p* = .20; breast *p* = .61; colorectal *p* = .94; data not shown).

In looking at the NCCN recommendations for the most common cancers (Table 2), the recommended frequency varied by tumor type and could be as infrequently as every 2–6 months for breast cancer that was treated with hormone therapy and 12 months for melanoma. Data on the frequency of tumor assessments in the real world is sparse. The real‐world frequency was more often than the frequency suggested by NCCN guidelines, but studies reporting these frequencies were conducted prior to the recent NCCN re‐evaluations of their guidelines for the most frequent cancer types.

**TABLE 2:** National Comprehensive Cancer Network recommendations^5^ and real‐world examples of frequency of tumor assessments in patients with metastatic or unresectable cancer, by cancer type and a comparison of frequency in clinical practice

Cancer type	NCCN frequency recommendations	Real‐world frequency
Non‐small cell lung cancer		
Initial CT scan findings: solid nodules or subsolid nodules with solitary ground glass nodules	6–12 months	The median number of imaging tests per patient in association with first‐line therapy varied by country, ranging between 3 (Brazil and Italy) and 14 (Japan)^6^ ~2 months (US)^7^
Initial CT scan findings: subsolid nodules with (solitary part solid or multiple subsolid)	3–6 months	
Breast		
Post‐chemotherapy	CT: Every 2–4 cycles Bone scan: Every 4 cycles	>4 imaging tests per year is considered “extreme use,” and is estimated to be 33% of US Medicare population^8^
Post‐ hormone	CT: Every 2–6 months Bone scan: Every 4–6 months	
Colorectal		
Locoregional	6–12 months	~3 months^9^
Metastatic	3–6 months	
Prostate	Bone Scan: For symptoms and as often as every 6–12 months	
Melanoma		
Uveal	Low risk: every 12 months; medium risk: every 6–12 months; high risk: every 3–6 months	~2 months^7^
Cutaneous	Frequency not indicated.	

## 4 DISCUSSION

In our examination of tumor scan frequency in oncology studies, we found that studies that assessed progression less frequently (i.e., longer intervals between progression scans) more often occurred in tumor types and settings where PFS was longer, either due to the natural biology of the disease or the treatment. This did not translate into significantly different PFS hazard ratios between categories of tumor assessment frequency. Several authors have suggested the possibility of biased PFS outcomes due to the timing of tumor assessment,^3^, ^10^ but in the studies that we included in our analysis, PFS outcomes of cases relative to controls were not differentially assessed between the tumor assessments frequency categories.

We should be careful to say that our study cannot exclude the possibility that the frequency of scanning can result in varying PFS hazard ratios. In all these trials, the sponsor and investigators chose the frequency with the knowledge of the underlying biology, and some prediction of the putative efficacy (this is required for a power calculation). That knowledge may lead to the choice of PFS assessment interval. Put another way, the PFS assessment interval is not randomly selected. As such, we cannot draw a firm causal conclusion that alternative intervals would not alter the observed hazard rations; Instead, we merely observe the phenomenon, that as conducted, our study failed to find such differences.

We also found that that median overall survival was longer in studies that assessed tumor frequency less often, which may be a result of less aggressive or slower growing tumors needing assessments less frequently. Because survival is a hard outcome, survival status should not be biased by treatment status like softer outcomes, such as PFS might be. One possible explanation for this is attrition bias or informative censoring from incomplete follow‐up when there are longer intervals between tumor assessments.^11^, ^12^ Conversely, studies that had a longer interval between assessments may have been those with an intervention that was less impactful on survival outcomes.

We were not able to find much data on the frequency of tumor assessments in the real world, and it was difficult to determine tumor assessment frequency in clinical practice compared to the frequency in clinical studies or in clinical guidelines. Future studies need to be done to better characterize tumor assessment frequency in real world practice. The frequency of scans in clinical studies was often more frequent than NCCN guidelines, but the recommended frequencies have recently been updated – suggesting less frequent tumor assessments.

### 4.1 Limitations

There are three limitations to our analysis. First, not all studies reported a PFS hazard ratio, which may have biased out results. As a sensitivity analysis, we also used a calculated risk ratio of median PFS for control participants and intervention participants, which resulted in similar results as results using the PFS hazard ratio. Second, our results may not be generalizable to all oncology studies since we used studies from only three oncology journals. While the number of journals was small, the journals are high‐impact journals that publish high‐quality publications on the larger oncology studies. Third, our categorization of tumor assessment frequency could not fully capture the multiple assessment frequencies used in some of the studies. In assigning a frequency category, we tried to select the frequency used for the longest duration, and the one in which progression was most likely to occur, as these were studies on metastatic tumors.

## 5 CONCLUSION

In conclusion, we document the frequency of scans in a range of contemporary randomized controlled trials. We found that less frequent tumor assessment frequency was associated with longer median PFS in both intervention and control groups of clinical oncology trials, but was not associated with differences in PFS hazard ratios. This may be explained by deliberate choices made by investigators to assess progression less frequently based on lower event rates. Future research is needed to compare real world to trial assessment.

## CONFLICT OF INTEREST

Dr. Prasad reports receiving royalties from his books, Ending Medical Reversal and Malignant; that his work is funded by the Arnold Ventures; that he has received honoraria for grand rounds/lectures from several universities, medical centers, and professional societies and payments for contributions to Medscape; Dr. Prasad hosts the podcast Plenary Session, which has Patreon backers. Dr. Haslam and Ms. Gill have no disclosures to report.

## AUTHOR CONTRIBUTIONS

**Alyson Haslam:** Data curation (lead); formal analysis (lead); methodology (equal). **Jennifer Gill:** Data curation (supporting); validation (supporting); writing‐review & editing (supporting). **Vinay Prasad:** Conceptualization (equal); funding acquisition (lead); methodology (equal); supervision (equal); writing‐review & editing (equal).

## ETHICS STATEMENT

All data were publicly available and non‐identifiable to patients or study participants, so no institutional review board approval was required, nor was individual informed consent.

## Data Availability

These data were derived from journal websites that are public domain.
